# Anæmic Coma

**Published:** 1849-07

**Authors:** 


					﻿Ancemic Coma, Hydren-cephaloid Affection, etc.—We had intended to
have prepared an article on the subject of the cerebral affection superven-
ing, in children especially, upon affections attended by innutrition and
protracted discharges, which often simulates encephalitis, and is not unfre-
quently considered and treated as inflammatory in its character. Having
found it impossible, on account of other duties, to carry out this intention,
we submit, in lieu of an original communication, the following extract from
the late excellent work on the brain by Samuel Solly, F. R. S., etc. The
subject is pne of no small importance, inasmuch as practitioners are apt to
differ in their views of the pathology of such cases, and, consequently, in
the appropriate measures of treatment. As the affection referred to frequent-
ly complicates the disease incident to the summer season, viz. cholera infan-
tum, the subject is particularly appropriate to the present time. We beg
to commend it to the careful consideration of our readers. We feel obliged
to omit several cases which are detailed in connection with the remarks
which follow, in consequence of the space which they would require.
Editor Buffalo Medical Journal.
Anaemic Coma.—We have seen that delirium may arise from an anaemic
condition of the brain, and we shall next observe that a state of insensibility
may be produced by similar causes.
I believe that if cerebral anaemia be allowed to continue for a long period,
it will occasionally terminate in hydrocephalus, and also in the white form
of ramollissement.
We will lirst consider anaemic hydrocephalus, with its effect, anaemic
coma, for I believe that there are two forms of hydrocephalus, the one
anaemic, the other inflammatory, as well as two forms of ramollissement.
Dr. Marshall Hall was one of the first to point out the resemblance which
exists between a comatose condition arising from exhaustion, and that
which is occasioned by inflammation and effusion. The affection which
Dr. Hall described, arises principally in infants, but it is not confined to
them. He calls it “ an hydrencephaloid affection of infants arising from
exhaustion.”
Dr. Hall has observed this affection generally as a consequence of contin-
ued diarrhoea, produced either by bad diet or long continued use of purga-
tive medicines, or as a consequence of blood-letting. He divides the affec-
tion into two stages, “ the first that of irritability, the second that of torpor;
in the former there appears to be a feeble attempt at re-action, in the latter
the nervous powers appear to be more prostrate.” He thus describes the
signs of complaint: “The infant becomes irritable, restless and feverish,
the face flushed, the surface hot, and the pulse frequent; there is an undue
sensitiveness of the nerves, and the little patient starts on being touched,
or from any sudden noise; there are sighing, moaning during sleep, and
screaming; the bowels are flatulent and loose, and the evacuations are
mucous and disordered. If, through an erroneous notion as to the nature
of this affection, nourishment and cordials be not given; or, if the diarrhoea
continue, either spontaneously or from the administration of medicine, the
exhaustion which ensues is apt to lead to a very different train of symptoms.
The countenance becomes pale, and the cheeks cool or cold; the eyelids
are half closed, the eyes are fixed, and unattracted by any object placed
before them, the pupils unmoved by the approach of light; the breathing,
from being quick, becomes irregular and affected by sighs; the voice becomes
husky; and there is sometimes a husky, teazing cough ; and eventually the
strength of the little patient has been subdued, and the vascular system
exhausted, by the abstraction of blood.”
Dr. Hall considers that this affection is to be distinguished from true
hydrocephalus principally “ by observing the condition of the countenance,
and by tracing the history and causes of the affection.
Dr. Abercrombie observes, “ in the last stages of diseases of exhaustion,
patients frequently fall into a state resembling coma, a considerable time
before death, and while the pulse can still be felt distinctly; I have many
times seen children lie for a day or two in this kind of stupor, and recover
under the use of wine and nourishment It is often scarcely to be distin-
guished from the coma which accompanies diseases of the brain. It attacks
them after some continuance of exhausting disease, such as tedious or
neglected diarrhoea, and the patients lie in a state of insensibility, the pupils
dilated, the eyes open and insensible, the face pale and the pulse feeble.—
It may continue for a day or two, and terminate favorably, or it may prove
fatal. This affection seems to correspond with the apoplexia ex inanitione
of the older writers. It differs from syncope by coming on gradually, and
in continuing a considerable time, perhaps a day or two; and it is not, like
syncope, induced by sudden and temporary causes, but by causes of grad-
ual exhaustion going on for a considerable time. It differs from mere
exhaustion, in the complete abolition of sense and motion, while the pulse
can be felt distinctly, and is, in some cases, of considerable strength. I
have seen in adults the same affection, though perhaps it is more uncommon
than in children.” In a letter which Dr. Hall received from Dr. Abercrom-
bie, that gentleman observes, “ The state of infants which I have referred
to, is a state of pure coma, scarcely distinguishable, at first sight, from the
perfect stupor of the very last stage of hydrocephalus, the child lying with
the eyes open, or half open, the pupils dilated, the face pale. It is difficult
to describe distinctly the appearance, but it is one which conveys the ex-
pression of coma, rather than of sinking; and I remember the first time I
met with the affection, the circumstance which arrested my attention, and
led me to suppose the disease was not hydrocephalus, the state somewhat
different from coma, was finding on further inquiry, that it came on after
diarrhoea, and not with any symptom indicating an affection of the head.—
The child recovered under the use of wine and nourishment.”
“ The remedies for this affection,” says Dr. Hall, “ are such as will check
this diarrhcea, and afterwards regulate the bowels, and restore and sustain
the strength of the little patient. With the first object, it may be necessary
to give the tinctura opii and chalk, and afterwards the pilula hydrargyri,
rhubarb and magnesia; with the second, sal volatile, but especially brandy,
and proper nourishment are to be given according to circumstances. But
in this, as in many cases of infantile disorders, the young milk of a young
and healthy nurse is the best remedy of all; in the absence of which
asses’ milk may be tried, but certainly not with the same confident hope of
benefit.”
“ Five or ten drops of the sal volatile may be given every three or four
hours, and twice, or thrice, in the interval five or ten drops of brandy may
be given in arrow-root done in water. As the diarrhoea and appearances
of exhaustion subside, these remedies are to be subtracted, the bowels are
to be watched and regulated, and the strength is to be continually sustained
by the nurse’s or asses’ milk. The brandy has sometimes appeared to
induce pain—sal volatile is then to be substituted for it; a dose of magne-
sia has also appeared to do good. For the state of irritability, the warm-
bath is a remedy of great efficacy. For the coma, a small blister or sina-
pism should be applied to the nape of the neck. A state of exhaustion of
the general system, as I have observed elsewhere, by no means precludes
the possibility of real congestion of the brain. It rather implies it. In
extreme cases these are not only the symptoms of cerebral congestion
during life, but effusion of serum into the ventricles of the brain is found
on examination after death. In every case the extremeties are to be kept
warm by flannel, and the circulation should be promoted in them by assi-
duous frictions. It is of the utmost importance carefully to avoid putting
the little patient into the erect posture. A free current of air is also a
restorative of the greatest efficacy.”
Dr. M. Hall follows up this account with some excellent cases very illus-
trative of his views; he also quotes the following observations of Dr. Gooch,
which, like all that this excellent practitioner ever penned, are worthy of
attention:—
“ So inveterate is the disposition to attribute drowsiness in children to
congestion of the brain, and to treat it so, that I have seen an infant four
months old, half dead from diarrhoea produced by artificial food, and capa-
ble of being saved only by cordials, aromatics, and a breast of milk; but
because it lay dozing on its nurse’s lap, two leeches had been put on the
temples, and this by a practitioner of more than average sense and knowl-
edge. I took off the leeches, Stopped the bleeding of the bites, and
attempted nothing but to restrain the diarrhoea, and get in plenty of nature’s
nutriment, and as I succeeded in this the drowsiness went off and the
child revived. If it could have have reasoned and spoken, it would have
told this practitioner how wrong he was; any one, who from long defect in
the organs of nutrition is reduced so that he has neither flesh on his body
nor blood in his veins, well knows what it is to lay down his head and doze
away half the day without any congestion or inflammation of the brain.—
This error, although I have specified it only in a particular complaint of
children, may be observed in our notions and treatment of other diseases,
and at other periods of life. If a woman has a profuse hemorrhage after
delivery, she will probably have a distressing headache, with throbbing in
the head, noises in the ears, a colorless complexion, and a quick, weak, often
thrilling pulse, all which symptoms are greatly increased by any exertion.
I have seen this state treated in various ways, by small opiates, gentle ape-
rients, and unstimulating nourishment, with no relief. I have seen blood
taken away from the head, and it has afforded relief for a few hours, but
then the headache, throbbing, and noises, have returned worse than ever;
the truth is, that this is the acute state of what in a minor degree, and in
a more chronic form, occurs in chlorosis, by which I mean pale-faced
amenorrhoea, whether at puberty or in after-life. It may be called acute
chlorosis, and like that disease, is best cured by steel, giv<m at first in small
doses, gradually increased, merely obviating constipation by aloe tic aperi-
ents.”
My esteemed friend and colleague, Dr. Risdon Bennett, in his admirable
work on Hydrocephalus, advocates the doctrine, that this disease assumes
very distinct forms; and that though it undoubtedly docs arise in some
instances from inflammation, in others it arises from an opposite condition.
He says,* “ There can be no difficulty in admitting that the physical alter-
ations of softening and serious effusion may be induced by functional and
organic changes, very different from inflammation or any allied morbid
action.” He considers that in by far the largest class of cases, the disease
is essentially the result of scrofulous action, and may or may not be atten-
ded by the signs of inflammation.
The comatose condition which we see occasionally following a severe
attack of erysipelas of the head in a debilitated constitution, come into this
category of antemic affections of the brain. It is difficult to say whether
this condition of the brain is the result of that general depression which is
both cause and effect in the erysipelas of London, or whether it can be
attributed to a derivation of blood from the surface. We all know that in
hyperaemia of the brain we can relieve our patients by determining the
blood to the surface. It is therefore possible, that this morbid cutaneous
determination of blood has the effect of diminishing the supply to the capil-
laries of the brain as effectually as our artificial measures. In a practical
point of view there is nothing more important to the surgeon than a knowl-
edge of the fact that a rambling, incoherent mania in the day, with a rest-
less delirium at night, is no proof of the existence of inflammatory action in
the brain.
It is indeed an interesting question to us, what is the positive state of
brain which accompanies this disorder of the intellect I believe that it is
a state of local amentia. It is not often that we have the opportunity of
examining it in its simple, uncomplicated condition, as is so freqently follows
an inflammatory state, though the inflammation may have been one of a
low or sub-acute character. With the view of ascertaining the condition of
the brain, 1 have always anxiously sought for every opportunity of exam-
ining the brain of patients who have died in this state, and I have generally
found it free from all signs of inflammation. Nevertheless, though it is true
that in London, as a general rule, erysipelas is a disease of debility, requi-
ring wine, brandy, &c., we must remember that acute inflammation of the
scalp will sometimes travel by continuity of tissue to the membranes of the
brain, and then prove rapidly fatal, either by serous effusion or by acute
inflammation of the hemispherical ganglion. We should therefore endea-
vor, in our constitutional treatment, to steer a middle course on the onset,
keeping, as sailors say, your weather eye open for a storm from very oppo-
site points of compass. A scruple of rhubarb and calomel, that is, five
grains of calomel to fifteen of rhubarb, followed by a senna draught in the
morning, clears the prima via and fits the system for either course, as sub-
sequent events may direct. In wounds of the scalp it may be well to re-
mark that often when the pericranium has been extensively separated from
the bone, and the edges of the wound heal quickly, there is always danger
* The Causes, Nature, Diagnosis, and Treatment of Acute Hvdiacephalus, 1848,
p. 147.
of subsequent suppuration, and the surgeon must be on the alert to make
a free opening, as matter cannot be pent up between the tendon of the
occipito frontalis and the bone without the brain sympathizing.
I remember inspecting the body of a very fine young woman who died
with erysipelas of the scalp, and in whom there was a large collection of
pus under the back part of the pericranium, which the medical man failed
to detect during life, but which most assuredly must have been instrumental
in producing the fatal result, for there was a corresponding inflammation of
the membranes of the brain, though not of an active character or great
extent; there was also some serous effusion.
A morbid condition of the brain of an ansemic character is often induced
in London by long-continued dyspepsia, with confinement in an impure
atmosphere.
I have frequently been consulted by professional men. and others engaged
in business in London, who, suffering from London cachexia, have supposed
that they have some disease of the brain. In one instance my patient sta-
ted that he had lost his memory, that he frequently would ring the bell and
forget before his servant answered it what he had rung it for; he complained
also, of having a sort of muzzy feeling in his head, that he could not read
or apply himself to anything; horse exercise was dreadful to him. He was
a long while in getting well, but it was ultimately effected, entirely by
means of very mild tonics.
Bakers are very liable to these affections, owing, I suppose, to their irre-
gular habits, sudden alternations of temperature, and disturbance of the
natural hours of rest, &c. I do not refer to the decidedly intemperate.
When these symptoms have las.ted some time, they are subdued with
difficulty, and require care. It is very necessary that the practitioner
should inform the sufferer of their obstinacy, otherwise he will become impa-
tient and dissatisfied. In the treatment of them, stimulants are prejudicial,
and the tonics must be very mild. Steel and quinine seldom answer at
first, and afterwards only in small doses. Strict diet, pure air, and exercise
without fatigue, are more important than medicine. I make it almost an
invariable rule to give an active purgative—calomel, &c., at night, haustus
senna in the morning; and 1 examine the evacuation before prescribing any
other medicine. If the liver is at fault, taraxacum, with small doses of sul-
phate of magnesia, and sulphuric acid, will be found more beneficial gene-
rally than mercury. This diseased condition cannot be removed by a coup
de main, and it is not advisable to say much to your patients regarding its
nature, for if you convince them that it arises from debility, they think they
have nothing to do but to eat, drink, and sleep, and thus get strong, and
then they will be well. On the other hand, you must relieve their minds
of the impression that it is allied to apoplexy, and depends on fulness of
the head or inflammation of the brain, which is generally their feeling, or,
otherwise, they will be anxious to be cupped, leeched, &c. If you have
sensible persons to deal with, you may explain the real rationale of your
treatment; if not, you must keep them in ignorance; but especially guard
against dropping one word about weakness, for all prefer eating and drink-
ing to taking physic; and they will think they understand all about it, and,
throwing physic to the dogs, feed themselves. Finding this fail, they imme-
diately conclude that you have mistaken the nature of their case, go to
somebody else, who perhaps orders a few leeches to the head, which some-
times relieve for a day or two, from the reaction which takesjplace, and this
confirms their opinion, until they again get worse.
The state of the nervous functions in a chlorotic female forms another
good illustration of the effect of anaemia on the brain. It is not necessary
to detail symptoms which are familiar to all.
How instructive it is to watch the gradual disappearance of the head-
aches, often most violent, under a judiciously managed course of steel medi-
cine. After the bowels have been freely opened and the tongue clean,
there is nothing equal to the old steel mixture with the compound decoc-
tion of aloes and aromatic confection, with a drachm of the spiritus myris-
tici; but the practitioner must not expect the headache to be removed
immediately.
				

